# Retraction: Genetic variation and diversity in 199 *Melilotus* accessions based on a combination of 5 DNA sequences

**DOI:** 10.1371/journal.pone.0230311

**Published:** 2020-03-05

**Authors:** The PLOS ONE Editors

Following publication of this article [1], concerns were raised about the contribution of this study in the context of previously published works.

An assessment carried out in consultation with members of the Editorial Board found overlap in the research question, approach, and datasets with articles previously published by some of the same authors [2, 3]. [2] was cited, but the relationship to this work was not clearly discussed. The previous work [2] investigated the phylogenetic relationships among *Melilotus* species by analysing DNA sequence data from four of the same loci: *rbc*L, *mat*K, *trn*L-F and ITS. The current study [1] uses sequence data from one additional region, *psb*A-*trn*H, and analyzes a larger dataset (the majority of additional accessions were from the National Gene Bank of Forage Germplasm (NGBFG, China)). The dataset for this study [1] reused some of the raw sequencing data used in [2] and [3].

The authors have stated that the emphasis of the articles differs, with [2] focusing on interspecific phylogenic relationships within genus *Melilotus*, and [3] focusing on constructing the standard barcode sequence for each species, while the current study [1] focuses on the genetic variation and diversity of *Melilotus* accessions collected in China.

The Data Availability statement for this article [1] states that all relevant data are within the paper and its Supporting Information files. However, the full raw sequencing data generated during this study were not deposited in an appropriate public repository at the time of publication. The available sequencing data have since been deposited at Genbank with the following accession numbers: 166 ITS sequences (MK918643—MK918756, MK918758—MK918771, MK918773—MK918810); 164 *mat*K sequences (MN532828—MN532973, MN532975—MN532982, MN532984—MN532993); 165 *rbc*L sequences (MN532693—MN532708, MN532710—MN532717, MN532719—MN532728, MN533354—MN533485); 164 *trn*H-*psb*A sequences (MN533090—MN533103, MN533105—MN533112, MN533114—MN533123, MN533222—MN533353); and 117 *trn*L-F sequences (MN533486—MN533501, MN533503—MN533510, MN533512—MN533521, MN533622—MN533688, MN533690—MN533705).

Eight germplasm accessions were omitted from the published S1 Table in error: PI549124, PI549125, PI549126, and PI549127 belonging to *M*. *albus*; ZM-2483 belonging to *M*. *dentatus*; and PI595329, PI595335 and PI595338 belonging to *M*. *officinalis*.

In light of the concerns about the overlap in the research question, approach, and datasets with previously published work, the *PLOS ONE* Editors retract this article.

HZ, FW, WG, RB, ZY, BPM, QY, YZ, XY and JZ did not agree with retraction.

## References

[1] Zhang H, Wu F, Guo W, Bai R, Yan Z, Muvunyi BP, et al (2018) Genetic variation and diversity in 199 *Melilotus* accessions based on a combination of 5 DNA sequences. PLoS ONE 13(3): e0194172 10.1371/journal.pone.0194172 29534094 PMC5849350

[2] Di H, Duan Z, Luo K, Zhang D, Wu F, Zhang J, et al (2015) Interspecific Phylogenic Relationships within Genus *Melilotus* Based on Nuclear and Chloroplast DNA. PLoS ONE 10(7): e0132596 10.1371/journal.pone.0132596 26167689 PMC4500581

[3] Wu F, Ma J, Meng Y, Zhang D, Pascal Muvunyi B, Luo K, et al (2017) Potential DNA barcodes for *Melilotus* species based on five single loci and their combinations. PLoS ONE 12(9): e0182693 10.1371/journal.pone.0182693 28910286 PMC5598934

